# The Dynamical Behavior of the s-Trioxane Radical Cation—A Low-Temperature EPR and Theoretical Study

**DOI:** 10.3390/molecules191117305

**Published:** 2014-10-28

**Authors:** Sergej S. Naumov, Wolfgang Knolle, Sergej P. Naumov, Andreas Pöppl, Igor Janovský

**Affiliations:** 1 Ingenieurgesellschaft Auto und Verkehr (IAV GmbH), Rockwellstraße 16, Gifhorn D-38518, Germany; E-Mail: dr.sergej.naumov@iav.de; 2 Leibniz-Institute for Surface Modification, Permoserstraße 15, Leipzig D-04318, Gemany; E-Mails: Sergej.Naumov@iom-leipzig.de (S.P.N.); igor.v.j@c-box.cz (I.J.); 3 Department of Physics, University of Leipzig, Linnéstraße 5, Leipzig D-04103, Gemany; E-Mail: poeppl@physik.uni-leipzig.de

**Keywords:** radical cations, s-trioxane, EPR, DFT, dynamical exchange

## Abstract

The radical cation of s-trioxane, radiolytically generated in a freon (CF_3_CCl_3_) matrix, was studied in the 10–140 K temperature region. Reversible changes of the EPR spectra were observed, arising from both ring puckering and ring inversion through the molecular plane. The ESREXN program based on the Liouville density matrix equation, allowing the treatment of dynamical exchange, has been used to analyze the experimental results. Two limiting conformer structures of the s-trioxane radical cation were taken into account, namely “rigid” half-boat and averaged planar ones, differing strongly in their electron distribution. The spectrum due to the “rigid” half-boat conformer can be observed only at very low (<60 K) temperatures, when the exchange of conformers is very slow. Two transition states for interconversion by puckering and ring-inversion were identified, close in activation energy (2.3 and 3.0 kJ/mol calculated). Since the energy difference is very small, both processes set on at a comparable temperature. In the case of nearly complete equilibration (fast exchange) between six energetically equivalent structures at T > 120 K in CF_3_CCl_3_, a septet due to six equivalent protons (hfs splitting constant 5.9 mT) is observed, characteristic of the dynamically averaged planar geometry of the radical cation. DFT quantum chemical calculations and spectral simulation including intramolecular dynamical exchange support the interpretation.

## 1. Introduction

The technique of low-temperature EPR spectroscopy in freon matrices is well established and has been used successfully to investigate a large variety of radical cations, generated radiolytically through positive charge transfer from matrix to solute. Radical cations of saturated six-membered rings with oxygen atoms have already attracted much attention [1,2,3,4], but the results obtained are sometimes contradictory, regarding both species assignment and structure. It is well known that a “rigid” form of these species can exist as a chair, boat or envelope conformation and the puckering motion has as a rule a relatively small activation energy for interconversion between different puckered structures. In the case of cyclic radical cations, besides interconversion through ring puckering inversion of the ring through the molecular plane (ring flip) was also observed [5,6,7].

The hyperfine structure (hfs) of EPR spectra associated with the radical cations, as well as the temperature dependence of their splitting patterns and line shapes can provide information, not only about the (rigid) molecular structure around the radical center, but also about molecular motion.

Although matrix interactions may influence the intrinsic dynamic behavior as well, the especially frozen CF_3_CCl_3_ matrix is known to have large internal cavities and weak solute-solvent interactions, being superior for the study of internal motional effects. Further, this matrix allows stabilization of radical cations up to 143 K, before the onset of a phase transition of the matrix.

In our previous report on s-trioxane and 1,3-dioxane radical cations [8] experimental data in the temperature range >77 K were presented. The aim of this work was to extend the measurements in CF_3_CCl_3_ down to 10 K, thus freezing the exchange between different conformers, and to discuss conformational changes of the s-trioxane radical cation on the basis of simulations involving the dynamic exchange between different conformers. Quantum chemical calculations were used to assist in identification of the involved species, defining possible transition states and calculation of hyperfine splitting constants and energy levels. The energy levels are calculated now with a larger basis set B3LYP/6-311+G(d,p), which produces more reliable results. The results are compared with the radical cation of 1,3-dioxane, as the latter has a similar electronic structure as the “frozen” radical cation of s-trioxane [8,9].

## 2. Results and Discussion

**Figure 1:**
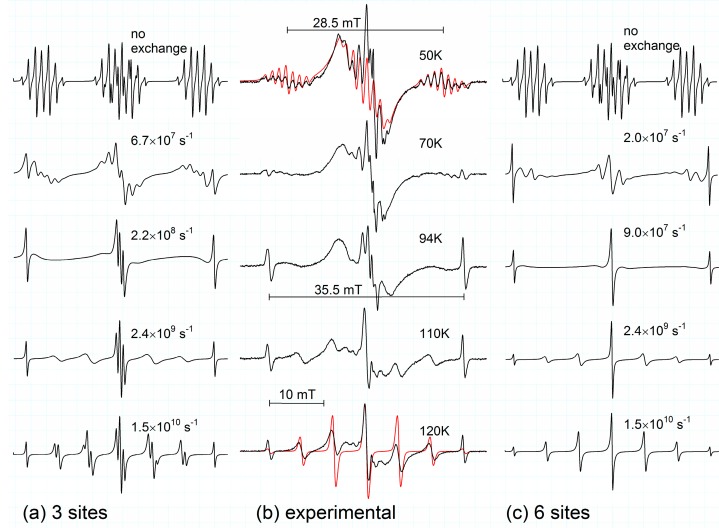
Calculated spectra taking into account the dynamical exchange between three equivalent sites (**a**) and six equivalent sites (**c**). Exchange rates as denoted. EPR-spectra (**b**) measured in irradiated frozen 0.1 mol % s-trioxane/CF_3_CCl_3_ solutions in the temperature range 50–120 K. Simulation of rigid half-boat and averaged planar structures shown in red, coupling constants given in Table 1.

For convenience, the experimental results obtained previously on the s-trioxane radical cation at T > 77 K are briefly summarized here [8]: The EPR spectra measured with 10^−1^ mol % s-trioxane in CF_3_CCl_3_ at 77–95 K have a total splitting of ~35.5 mT and show a very strong line broadening in the central part (*cf.* Figure 1b, spectrum at 94 K, remembering that the broad anisotropic background is due to freon radicals). By increasing the temperature new lines appear in the spectrum and at 120 K eventually a seven-line spectrum (“septet”) is observed (Figure 1b). This change was reversible, but at temperatures >120 K further irreversible changes in the spectrum occur, most probably due to the formation of radicals. The spectra measured with 10^−1^ mol % s-trioxane at 77 K in CF_2_ClCFCl_2_ have the same total width as in CF_3_CCl_3_, are somewhat better resolved and also show a strong line broadening in the central part. When the temperature is increased to 94 K a fast transformation into a broad singlet (∆H_pp_ = 1.7 mT) occurs, which was assigned to an apparent deprotonated s-trioxanyl radical [10,11]. Further details can be found in [8].

**Figure 2:**
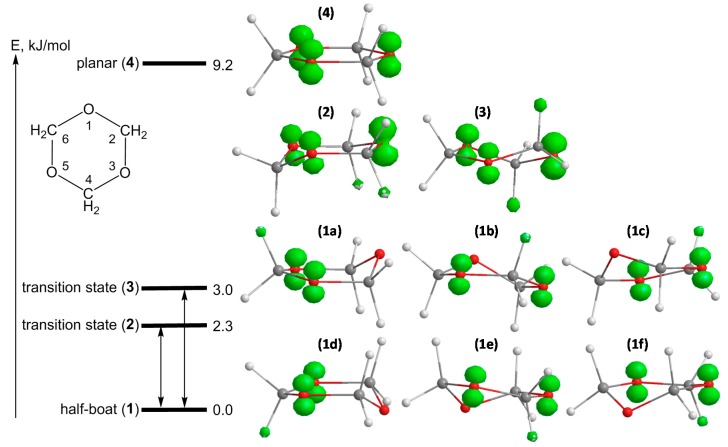
Energy scheme, geometrical structures and spin density distributions for different conformers of the s-trioxane radical cation: six stable half-boat conformers **1a**–**f**, transition structures for ring inversion **2** (chair) and for ring puckering **3** (twisted boat) and the averaged planar geometry **4** (saddle point). Calculated at B3LYP/6-311+G(d,p) level of theory.

Extending the temperature range and cooling the samples down to 10 K, a well resolved spectrum appears, which is roughly a triplet of a multiplet (seven lines) (*cf. *Figure 1b). There are no significant changes of the spectra in the range of 10–50 K, thus the spectrum at 50 K is given here as a reference due to a slightly better resolution (additional line broadening at lower temperature). Spectral changes between 10–120 K are completely reversible. The coupling constants determined in CF_3_CCl_3_ matrix at T = 50 K are *a*/mT = 14.9 (1H), 13.3 (1H), 2.36 (2H) and 1.36 (2H). They agree well with the data published by Rhodes and Symons at 30 K in CFCl_3_ matrix: *a*/mT = 14.2 (2H, only an averaged value was given), 2.5 (2H) and 1.3 (2H) [3]. QC calculations for the conformer with lowest energy show that the radical cation has a preferred half-boat conformation (*cf. *Figure 2, structures **1a**–**f**) with the spin distributed between two oxygen centers (note that the preferred original configuration of the neutral molecule is the chair one). The two protons of the methylene group embedded between the oxygen atoms bearing the spin are magnetically not equivalent due to a small tilt, *i.e*., the general half-boat conformation is not exactly “flat” (the dihedral angle is calculated to be ~12°). The measured coupling constants and calculated parameters are summarized in Table 1.

**Table 1:** Calculated values of the atomic spin densities ρ and of isotropic hfs splitting constants *a*(H)/mT for structures **1**–**4** of the s-trioxane radical cation (numbering of atoms is given in Figure 2). Hfs splitting constants, determined in CF_3_CCl_3_ matrix and assigned to the rigid structure **1** (at 50 K) and to the averaged structure **4** (at 120 K) are given for comparison.

Structure	Parameter	Atom					
		O1	C2	O3	C4	O5	C6
**1** (half boat)	ρ _B3LYP_	0.294	−0.011	0.294	−0.013	0.056	−0.013
	*a*(H) _B3LYP_		13.1/8.8		4.24/2.18		4.24/2.18
	*a*(H) _BH&HLYP_		17.8/12.4		2.46/1.45		2.46/1.45
	*a*(H) _exp.,50K_		14.9/13.3		2.36/1.31		2.36/1.31
**2** (chair)	ρ _B3LYP_	0.277	−0.014	0.178	−0.018	0.167	−0.014
	*a*(H) _B3LYP_		8.6/5.4		8.2/5.0		6.3/2.1
**3** (twisted boat)	ρ _B3LYP_	0.189	−0.007	0.181	−0.013	0.267	−0.013
	*a*(H) _B3LYP_		5.0/5.0		9.2/3.1		9.4/3.4
**4** (planar)	ρ _B3LYP_	0.294	−0.011	0.294	−0.013	0.056	−0.013
	*a*(H) _B3LYP_		6.6/6.6		6.6/6.6		6.6/6.6
	*a*(H) _BH&HLYP_		6.9/6.9		6.9/6.9		6.9/6.9
	*a*(H) _exp.,120K_		5.9/5.9		5.9/5.9		5.9/5.9

**Figure 3:**
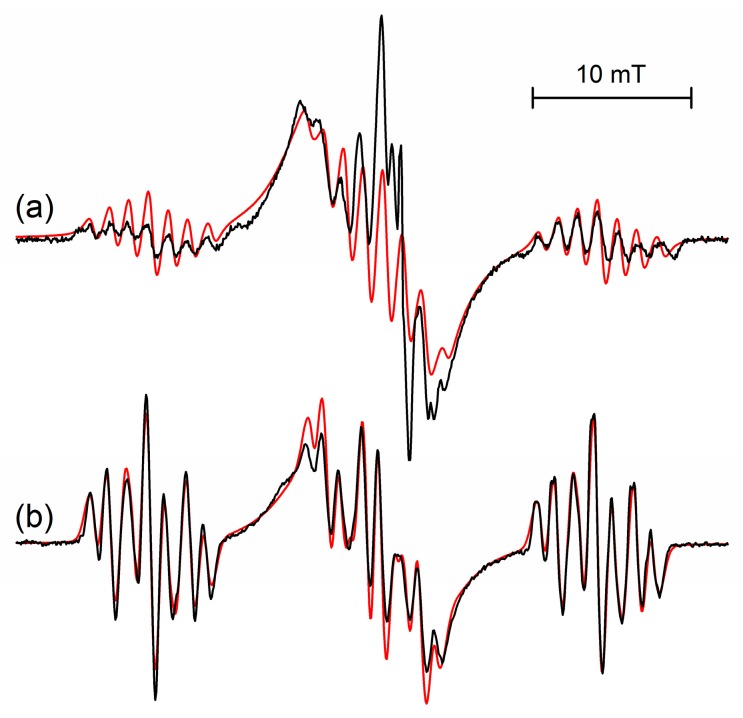
Comparison of the EPR spectra of the radical cations of (**a**) s-trioxane at 50 K and (**b**) 1,3-dioxane at 95 K observed after irradiation of 0.1 mol% CF_3_CCl_3_ solutions. Spectral simulations are shown with red lines. Parameters of s-trioxane are given in Table 1 (50 K), data for 1,3-dioxane are *a*(H)/mT = 14.6 (1H), 13.7 (1H), 2.45 (2H), 1.0 (2H).

The structure of the frozen rigid conformation of the s-trioxane radical cation is comparable to the structure of the radical cation of 1,3-dioxane (*cf.* Figure 3a,b), for which coupling constants *a*(H)/mT of 14.6, 13.7, 2.45 (2H) and 1.0 (2H) were determined [1,2,8]. With respect to energetically (and magnetically) equivalent conformers of both, the radical cation of s-trioxane—owing to its high symmetry—has six equivalent conformers of its low-energy half-boat structure (*cf.* Figure 2, **1a**–**f**) whereas 1,3-dioxane has only two possible equivalent structures.

In the case of s-trioxane at high temperatures obviously interconversion of equivalent structures occurs. The question is how does this interconversion proceed (by rotational and/or ring flipping modes), if rotational ring puckering involving only conformers with oxygen puckered on the same side of the molecular plane can be distinguished from interconversion of all six conformers and the energetical pathway for interconversion were addressed by quantum chemical means and by comparing experimental spectra with those calculated including dynamical exchange.

The geometry optimizations and the vibrational analysis for the s-trioxane radical cation (data for spin density distributions and hfs coupling constants are summarized in Table 1) yield two stationary points on the potential energy surface, which are half-boat (Figure 2, structure **1**) and planar (structure **4**) conformations, the planar form being less stable at 77 K by 9.2 kJ/mol (*cf.* also [8]). The frequency calculation on the half-boat conformation doesn’t produce negative frequencies, indicating that this structure is truly the minimum. The frequency analysis for planar structure **4** yields three imaginary frequencies, indicating that this structure is a third-order saddle point. The thorough optimization of transition states for half-boat to half-boat interconversion reveals now a second chair structure, in addition to the twisted boat recognized in our previous paper [8]. Both structures show only one imaginary frequency and have slightly different energies: 2.3 kJ/mol and 3.0 kJ/mol (at 77 K) for the chair (Figure 2, structure **2**) and the twisted boat (Figure 2, structure **3**) conformers, respectively. The analysis of the vibrations related to the negative frequencies reveals that transition structure **2** (chair) mediates ring inversion (ring flip) and twisted-boat structure **3** mediates ring puckering interconversion. Both potential energy barriers separating two minima are very small and comparable to the first deformational vibration level of the stable half-boat structure (97 cm^−1^ ~1.1 kJ/mol at B3LYP/6-311+G(d,p) level). As a consequence, the interconversion can proceed even at quite low temperatures, with an exchange rate depending on the temperature. It is also expected that such a small difference may render a distinction between ring inversion and puckering motions impossible.

Three models for interconversion were considered. Simulations of exchange broadening were made with the program ESREXN [12] using the formula derived by Norris [13] and taking hfs coupling constants derived from the experimental spectrum at 50 K and a line width of 0.2 mT as input. The first model took into account ring inversion between two sites only (ring flip via transition state **2**). This possibility could be ruled out quickly. The simulation of the dynamical exchange between two ring-inversed configurations discloses two strong side lines with a total separation of ~28.5 mT, approximately located in the center of the side groups (see Figure 1b, 50 K). This, however, is not observed; the spectra at 70 and 94 K in Figure 1b display clearly lines with a separation of ~35.5 mT. The second model took into account the pseudo-rotational puckering of the half-boat structure, the puckered oxygen being always on the same site of the molecular plane, thus exchange between three conformers was assumed. The third model included all six equivalent conformers (*cf.* Figure 2, **1a**–**f**). For all models only one exchange rate was considered, as the calculated transition state structures **2** and **3** are close in energy. For the model with six equivalent sites it is assumed that transformations between each two sites occur with the same probability. The results for model 2 and 3 are presented in Figure 1 side-by-side with typical experimental spectra. It is obvious, that the 3-site model can’t account for the septet with nearly binomial intensities observed at 120 K. At this temperature interconversion between all six conformers (fast exchange) occurs and the experimental spectrum at 120 K (Figure 1b) has to be explained by coupling of six equivalent protons, the experimentally derived hfs splitting constant of 5.9 mT (6H) being close to the value calculated for the planar structure **4** (see Table 1).

Comparing spectra in the temperature range 60–110 K with both simulations, there is a slight impression from the central part of the spectra and their outer wings, that the 3-site jump contributes initially more to the observed spectrum before dynamical line-broadening reaches its maximum effect. However, as most significant changes occur in a very narrow range of 65–77 K, and as the central part also contains contributions from stable freon radicals, it was impossible to distinguish any different onset of puckering and ring flipping interconversions. Also second-order contributions in the central part, typical for large hyperfine splittings [14], can’t be ruled out completely.

Definitely, ring inversion occurs significantly at 77 K (for spectra *cf.* [8]), thus one has to assume comparable rate constants. This is in contrast to the case of the c-pentane radical cation where spectral changes in the temperature interval 62–83 K could clearly be attributed to a preferable ring inversion, whereas ring puckering contributed significantly to the changes observed in the range 89–108 K [6]. In that case activation energies where determined to be 4.9 kJ/mol and 15.2 kJ/mol, respectively, making a separation of both interconversion reactions possible. In the present case the barriers of 2.3 and 3.0 kJ/mol calculated for the transition states for puckering and inversion are too close to determine separate activation energies. The Arrhenius plot in the temperature interval 60–110 K using the estimated exchange rates (*cf.* Figure 1c) yields an experimental value for the activation energy of ~4.5 ± 0.5 kJ/mol, close to the calculated values and comparable to the value of 4.9 kJ/mol for ring inversion in case of c-pentane radical cation.

For 1,3-dioxane no dynamic effects where observed before the radical cations disappears at T ~110 K in CF_3_CCl_3_ [8]. Calculations show that the single twisted-boat transition state has an activation energy of 14.6 kJ/mol, five times higher than those for the transition states for s-trioxane. Thus, the activation energy is too high for an interconversion of 1,3-dioxane radical cation to be observable before decomposition at ~94 K occurs.

## 3. Experimental Section

The EPR experiments were performed using a Bruker 300E spectrometer (9.5 GHz, 100 kHz modulation, Bruker BioSpin GmbH, Rheinstetten, Germany) equipped with either a finger Dewar (77 K) or a ER 4121 VT variable temperature control unit (above 95 K). Spectra were recorded at a microwave power of 0.1 mW and modulation amplitude of 0.05 or 0.1 mT. Experiments below 77 K were performed with an ESR900 cryostat (Oxford Instruments, Abingdon, UK) and spectra were recorded at a microwave power of 5 µW (10–30 K) and 50 µW (>50 K) and a modulation amplitude of 0.1 mT. The WinSim software [15] was used for spectra simulation.

1,1,1-Trichlorotrifluoroethane (99%, Sigma-Aldrich, Munich, Germany or Merck, Darmstadt, Germany) was purified by passing it through a column filled with neutral alumina. 1,3-Dioxane (97%, Acros Organics (Thermo Fisher Scientific), Geel, Belgium), 1,3,5-trioxane (98%, Lancaster, Morecombe, UK), and 1,1,2-trichlorotrifluoroethane (Uvasol, Merck, Darmstadt, Germany) were used as supplied. Samples were always irradiated at 77 K. Further details on sample preparation and irradiation with the 10-MeV electron beam of a Linac are described elsewhere [16]. 

Quantum chemical calculations were performed using the Gaussian 03 [17] and Jaguar 8.3 [18] programs. Molecular and electronic structures of transients were investigated by the DFT B3LYP [19,20] functional and compared with BH&HLYP [21], because B3LYP tends to “overcorrelate” electrons in radical cations at geometries where localization of the unpaired electron should occur [22]. Activation energy (height of interconversion barrier between different puckered structures) and relative energies of the different conformers were calculated at B3LYP/6-311+G(d,p) level of theory. The frequency calculations were done at the same level of theory and were used to locate transition state geometries. Simulations of exchange broadening were made with the program ESREXN [12]. ESREXN provides two methods to calculate ESR exchange-broadened spectra: by use of the formula derived by Heinzer [12] or by the one derived by Norris [13]. Both formulas are equivalent, but Heinzer’s formula is faster in computation for the exchange of a lower number of conformations. Here, Norris’ equation was used to simulate the s-trioxane radical cation due to the higher number of conformers. Also, the computation with Heinzer’s formula caused a hang off of the computational procedure in case of s-trioxane. Source code is written with some older FORTRAN version (FORTRAN 66 or earlier) and need to be adapted to modern PC architecture.

## 4. Conclusions

In conclusion, the dynamic behavior of the s-trioxane radical cation stabilized in the temperature range 10–120 K in CF_3_CCl_3_ could be well explained on the basis of quantum chemical calculations and spectral simulations. The calculations predict only one stable half-boat structure for the radical cation of s-trioxane and two low-lying transition states of comparable activation energy for the ring puckering and ring inversion motions. Spectral simulations taking dynamical exchange into account reproduce the observed spectra reasonably well, if the exchange of all six energetically equivalent conformers is taken into account. When comparing the results of calculation of hfs splitting constants for stable structures of radical cations, the BH&HLYP/6-31G(d) method shows slightly better agreement with the experimental results than B3LYP/6-31G(d).

## References

[1] Snow L.D., Wang J.T., Williams F. (1982). Delocalized Pi-Radical Cations of Acetals. J. Am. Chem. Soc..

[2] Symons M.C.R., Wren B.W. (1984). Electron spin resonance spectra of ether radical cations generated by pulse radiolysis. J. Chem. Soc. Perkin Trans. 2.

[3] Rhodes C.J., Symons M.C.R. (1988). The Radical Cation of Formaldehyde in a Freon Matrix—An Electron-Spin Resonance Study. J. Chem. Soc. Faraday Trans. 1.

[4] Bonazzola L., Michaut J.P., Roncin J. (1994). Gamma-Irradiated Tetrahydropyran in Freon Matrices—Radical-Cation or Neutral Radical with a Heteronuclear Sigma-Asterisk C-O Bond. New J. Chem..

[5] Sjöqvist L., Benetis N.P., Lund A., Maruani J. (1991). Intramolecular dynamics of the C_4_H_8_NH radical cation—An application of the anisotropic exchange theory for powder ESR lineshapes. Chem. Phys..

[6] Sjöqvist L., Lund A., Maruani J. (1988). An Electron-Spin-Resonance Investigation of the Dynamical Behaviour of the Cyclopentane Cation in CF_3_CCl_3_. Chem. Phys..

[7] Lindgren M., Shiotani M., Lund A., Shiotani M. (1991). ESR studies of radical cations of cycloalcanes and saturated heterocycles. Radical Ionic Systems.

[8] Janovský I., Naumov S., Knolle W., Mehnert R. (2003). Investigation of the molecular structure of radical cation of s-trioxane: Quantum chemical calculations and low-temperature EPR results. Radiat. Phys. Chem..

[9] Glidewell C. (1983). Semi-Empirical Scf-Mo Study of the Molecular and Electronic-Structures of the Cation-Radicals Derived from Simple Ethers and Acetals. J. Chem. Soc. Perkin Trans. 2.

[10] Baranova I.A., Belevskii V.N., Feldman V.I. (1990). Electron-Spin-Resonance Studies of Ion-Molecular Reactions, Fragmentations and Rearrangements of Radical Cations and Neutral Radicals from the Cyclic Acetals as Studied. Vestn. Mosk. Univ. Khimiya.

[11] Dobbs A.J., Gilbert B.C., Norman R.O.C. (1971). Electron Spin Resonance Studies. Part XXVII. The Geometry of Oxygen-Substituted Alkyl Radicals. J. Chem. Soc. A.

[12] Heinzer J. (1971). Fast Computation of Exchange-Broadened Isotropic ESR Spectra. Mol. Phys..

[13] Norris J.R. (1967). Rapid computation of magnetic resonance line shapes for exchange among many sites. Chem. Phys. Lett..

[14] Weil J.A., Bolton J.R., Wertz J.E. (1994). Electron Paramagnetic Resonance. Elementary Theory and Practical Applications.

[15] Duling D.R. (1994). Simulation of multiple isotropic spin-trap EPR spectra. J. Magn. Reson. Ser. B.

[16] Knolle W., Janovský I., Naumov S., Mehnert R. (1999). Low-temperature EPR study of radical cations of 2,5- and 2,3-dihydrofuran and their transformations in freon matrices. J. Chem. Soc. Perkin Trans. 2.

[17] Frisch M.J., Trucks G.W., Schlegel H.B., Scuseria G.E., Robb M.A., Cheeseman J.R., Montgomery J.J.A., Vreven T., Kudin K.N., Burant J.C. (2004). Gaussian 03.

[18] (2014). Jaguar.

[19] Becke A.D. (1993). Density-functional thermochemistry III. The role of exact exchange. J. Chem. Phys..

[20] Becke A.D. (1996). Density-functional thermochemistry IV. A new dynamical correlation functional and implications for exact exchange-mixing. J. Chem. Phys..

[21] Becke A.D. (1993). A New Mixing of Hartree-Fock and Local Density-Functional Theories. J. Chem. Phys..

[22] Bally T., Borden W.T. (2007). Calculations on Open-Shell Molecules: A Beginner’s Guide. Reviews in Computational Chemistry.

